# Bidirectional Tumor-Promoting Activities of Macrophage Ezrin

**DOI:** 10.3390/ijms21207716

**Published:** 2020-10-19

**Authors:** Krishnendu Khan, Briana Long, Gauravi M. Deshpande, Paul L. Fox

**Affiliations:** 1 Department of Cardiovascular and Metabolic Sciences, Lerner Research Institute, Cleveland Clinic Foundation, Cleveland, OH 44195, USA; khank3@ccf.org (K.K.); longb@ccf.org (B.L.); 2 Lerner Research Institute Imaging Core, Cleveland Clinic Foundation, Cleveland, OH 44195, USA; deshpag@ccf.org

**Keywords:** ezrin, ITGAM, CD11b, MMP-9, VEGF-A, tumor-associated macrophages, tumor microenvironment, macrophage polarization, breast cancer

## Abstract

Ezrin links the cytoskeleton to cell surface integrins and plasma membrane receptors, contributing to the proliferative and metastatic potential of cancer cells. Elevated ezrin expression in several cancers is associated with poor outcomes. Tumor cell ezrin expression and function have been investigated in depth; however, its role in macrophages and other tumor microenvironment cells remains unexplored. Macrophages profoundly influence tumorigenesis, and here we explore ezrin’s influence on tumor-promoting macrophage functions. Ezrin knockdown in THP-1 macrophages reveals its important contribution to adhesion to endothelial cells. Unexpectedly, ezrin is essential for the basal and breast cancer cell-stimulated THP-1 expression of *ITGAM* mRNA that encodes integrin CD11b, critical for cell adhesion. Ezrin skews the differentiation of THP-1 macrophages towards the pro-tumorigenic, M2 subtype, as shown by the reduced expression of *FN1*, *IL10*, and *CCL22* mRNAs following ezrin knockdown. Additionally, macrophage ezrin contributes to the secretion of factors that stimulate tumor cell migration, invasion, and clonogenic growth. Lastly, THP-1 ezrin is critical for the expression of mRNAs encoding vascular endothelial growth factor (VEGF)-A and matrix metalloproteinase (MMP)-9, consistent with pro-tumorigenic function. Collectively, our results provide insight into ezrin’s role in tumorigenesis, revealing a bidirectional interaction between tumor-associated macrophages and tumor cells, and suggest myeloid cell ezrin as a target for therapeutic intervention against cancer.

## 1. Introduction

Ezrin is a member of the ezrin-radixin-moesin (ERM) family of cytoplasmic proteins whose primary function is connecting the plasma membrane to the underlying cortical actin cytoskeleton [1]. Ezrin shares substantial sequence similarity with other ERM proteins, i.e., moesin and radixin. The N-termini of ERM proteins harbor a ~300-amino acid FERM (four point one and ERM) domain that forms a cloverleaf-like structure; likewise, the last 38 amino acids in the C-terminal “tail” domain share limited sequence homology [2]. Ezrin is generally present in a dormant, non-phosphorylated state in which a closed conformation is established by the interaction of the N-terminal FERM domain with the C-terminal tail [3]. Following stimulus-dependent binding of the FERM domain to plasma membrane PIP_2,_ ezrin Thr^567^ phosphorylation unmasks the C-terminus to permit interaction with underlying F-actin [4]. Ezrin transduces extracellular signals by FERM domain interaction with trans-membrane proteins, including CD43, CD44, ICAM1, ICAM2, and EBP50 [5,6,7,8]. Ezrin also provides membrane tension and contributes importantly to cell shape regulation, proliferation, migration, and adhesion [9,10,11]. Elevated ezrin expression and abnormal cytoplasmic localization are associated with aggressive cancers, and positively associated with a degree of malignancy and poor prognosis in several cancer types [12,13,14]. Moreover, ezrin contributes to breast cancer stem cell chemo-resistance, and has been proposed as a prognostic marker [15]. By stimulating the epithelial-to-mesenchymal transition, ezrin is thought to promote tumor metastasis in breast and osteosarcoma cancer cells [16,17].

The tumor microenvironment includes multiple immune cell types, and plays a critical role in tumor initiation, progression, and metastasis [18,19]. Macrophages, generally abundant in the tumor microenvironment, can be classified in two major sub-groups, namely, classically activated M1 macrophages which exhibit anti-tumor activities, and alternatively activated, pro-tumorigenic M2 macrophages, also known as tumor-associated macrophages [20,21]. In co-culture studies, tumor-associated macrophages promote the proliferation, invasion, and metastatic and angiogenic potential of cancer cells, consistent with their stimulatory function in cancer development and progression [22,23,24,25,26]. Moreover, cancer cells can reprogram macrophages towards the M2 subtype, forming a feed-forward loop driving cancer progression [27,28]. Based on in vitro and in vivo studies revealing their important contribution to tumor progression, tumor-associated macrophages are potential therapeutic targets in mammary and other cancers [29,30,31].

Studies of the role of ezrin in cancer have focused primarily on cancer cells themselves, whereas its role in tumor microenvironment-resident or infiltrating cells remains largely unknown. Here, we explore the role of ezrin in macrophage function and interaction with cancer cells primarily using THP-1 monocytic cells as a model system, in conjunction with weakly aggressive MCF-7 and highly aggressive MDA-MB-231 breast cancer cell lines. As a surrogate for the investigation of monocyte homing to tumor-initiating sites, we determined the influence of myeloid cell ezrin on endothelial cell adhesion and the cancer cell-directed migration of THP-1 cells. We further determined the role of ezrin in macrophage polarization, and communication between macrophages and tumor cells. Our results illuminate mechanisms by which ezrin contributes to macrophage differentiation to tumor-associated macrophages within the tumor microenvironment, and re-directs its function toward pro-tumorigenic and pro-metastatic properties fundamental to cancer progression.

## 2. Results

### 2.1. Macrophage Ezrin Contributes to Adhesion and Migration towards Cancer Cells

**Figure 1:**
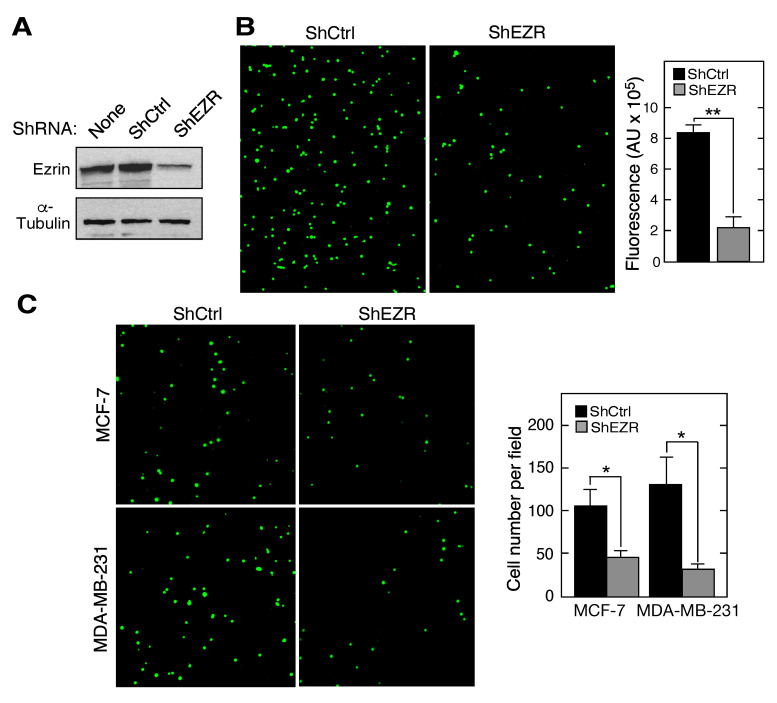
Role of myeloid cell ezrin in adhesion to endothelial cells and transmigration. (**A**) Immunoblot of lysates from THP-1 cells subjected to lentivirus-mediated stable knockdown with shRNA targeting ezrin (ShEZR) or control shRNA (ShCtrl). (**B**) Calcein-AM-labeled ShCtrl and ShEZR THP-1 cells were incubated with human umbilical vein endothelial cells (HUVEC) for 3 h and cell adhesion was determined by immunofluorescence imaging (left) and by the quantification of fluorescence intensity (right). (**C**) The chemoattraction of calcein-AM-labeled ShCtrl and ShEZR THP-1 cells towards conditioned medium (CM) from breast cancer cell lines was determined by a trans-well migration assay. Mean ± standard deviation; * and ** indicate *p* < 0.05 and 0.01, respectively.

Ezrin exhibits an adaptor function, facilitating the presentation of multiple cell surface proteins involved in cell-cell interactions [32,33]. An early step in tumorigenesis is the secretion of soluble chemoattractants by tumor cells that induce the binding of circulating CD11b+ myeloid cells to the vascular endothelium, an event necessary for diapedesis through the vessel wall and into the tumor microenvironment [34]. The role of myeloid cell ezrin in the interaction with endothelial cells was investigated using monocytic THP-1 cells subjected to lentivirus-mediated, shRNA-directed stable knockdown of ezrin (ShEZR), followed by labeling with calcein-AM. The highly efficient knockdown of ezrin in a stable cell line expressing ShEZR, but not in cells expressing scrambled shRNA (ShCtrl), was observed (Figure 1A). Ezrin knockdown reduced THP-1 binding to a confluent monolayer of human umbilical vein cells (HUVECs) by about 75% as shown by immunofluorescence imaging (Figure 1B, left) and by the quantification of fluorescence intensity (Figure 1B, right). The role of macrophage ezrin in chemoattraction towards breast cancer cells was determined by trans-well migration assay. Compared to ShCtrl cells, calcein-AM-labeled ShEZR cells exhibited markedly reduced transmigration toward conditioned medium (CM) generated by both MCF-7 and MDA-MB-231 cells as shown by imaging (Figure 1C, left) and by the quantification of migrating cell numbers (Figure 1C, right). These results are consistent with a pro-tumorigenic function of macrophage ezrin in which it facilitates cell interaction with vascular endothelium and the tumor-directed transmigration of macrophages. However, depending on the polarization state, tumor-infiltrating macrophages also can exhibit pro-inflammatory, anti-tumorigenic activities, as considered below, and thus macrophage ezrin might exhibit anti-tumor activity in some circumstances [35,36].

### 2.2. Role of Ezrin in Leukocyte Expression of Chemokine Receptors, Integrins, and Cell Surface Adhesion Molecules

**Figure 2:**
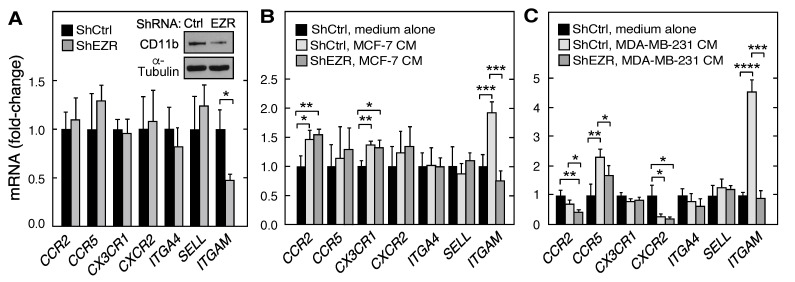
Role of myeloid cell ezrin in the basal and stimulated expression of leukocyte chemokine receptors, integrins, and cell surface adhesion molecules. (**A**) mRNAs encoding leukocyte cell surface proteins in ShEZR THP-1 cells were determined by RT-qPCR and normalized to ShCtrl cell mRNA; (inset) immunoblot analysis of CD11b and α-tubulin. (**B**,**C**) ShEZR and ShCtrl cells were incubated with CM from MCF-7 (**B**) and MDA-MB-231 (**C**) cells, or with medium alone and mRNAs encoding leukocyte surface proteins determined by RT-qPCR. Mean ± standard deviation; *, **, ***, and **** indicate *p* < 0.05, 0.01, 0.001, and 0.0001, respectively.

Leukocytes express the cell surface chemokine receptors, integrins, and adhesion molecules that contribute to migration and adhesion. The requirement for ezrin in the basal and stimulus-dependent expression of mRNAs of several key cell surface proteins in THP-1 cells was investigated by RT-quantitative PCR (qPCR). Of the mRNAs encoding leukocyte chemokine receptors, integrins, and cell surface adhesion molecules investigated, namely, *CCR2* (C-C motif chemokine receptor (CR) 2), *CCR5* (C-C motif CR 5), *CX3CR1* (C-X3-C motif CR 1), *CXCR2* (C-X-C CR 2), *ITGA4* (integrin α4), *SELL* (L-selectin), and *ITGAM* (integrin αM, CD11b), basal expression of *ITGAM* mRNA was uniquely influenced by ezrin depletion, exhibiting a reduction of about 50% (Figure 2A). Co-culture of macrophages with CM from both breast cancer cell lines markedly enhanced *ITGAM* mRNA expression; the stimulation by CM from the more aggressive MDA-MB-231 cells was about twice that by MCF-7 CM (Figure 2B,C). Remarkably, the CM-mediated stimulation of *ITGAM* mRNA expression in both cell lines was ezrin-dependent and completely suppressed by ezrin knockdown. The responses of the other genes to CM and ezrin knockdown were less dramatic or absent. The *ITGAM* gene encodes CD11b which partners with CD18 to form the β2 integrin Mac-1 on leukocyte cell surfaces, essential for the arrest and firm adhesion to the endothelium [37]. These results suggest that the ezrin-mediated induction of CD11b contributes importantly to myeloid cell adhesion to endothelial cells (EC). Immunoblot analysis confirmed the reduced expression of the *ITGAM* mRNA product, CD11b (Figure 2A, inset). Interestingly, the knockdown of moesin, an ezrin homolog, by shRNA targeting moesin (shMSN; Figure S1A, left) did not influence the THP-1 cell expression of ITGAM mRNA (Figure S1A, center) or CD11b (Figure S1A, right). These results are suggestive of the differential regulation of gene expression by FERM proteins.

### 2.3. Contribution of Ezrin to Macrophage Polarization

**Figure 3:**
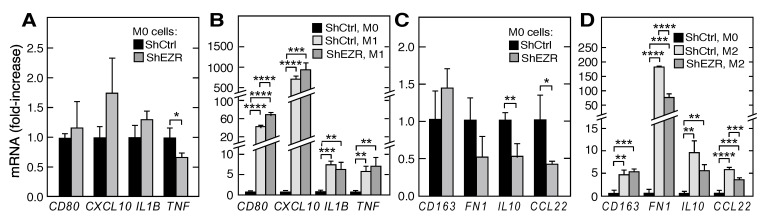
Role of ezrin in myeloid cell polarization. (**A**) mRNAs encoding M1 marker proteins *CD80*, *CXCL10*, *IL1B*, and *TNF* were determined by RT-qPCR in M0 ShEZR cells and normalized to M0 ShCtrl cells. (**B**) ShEZR cells and ShCtrl were subjected to M1 polarization conditions, and mRNAs encoding M1 marker proteins determined as in (**A**) and normalized to M0 ShCtrl cells. (**C**) mRNAs encoding M2 marker proteins *CD163*, *FN1*, *IL10*, and *CCL22* were determined by RT-qPCR in M0 ShEZR cells and normalized to M0 ShCtrl cells. (**D**) ShEZR and ShCtrl cells were subjected to M2 polarization conditions and mRNAs encoding M2 markers determined as in (**C**) and normalized to M0 ShCtrl cells. Mean ± standard deviation; *, **, ***, and **** indicate *p* < 0.05, 0.01, 0.001, and 0.0001, respectively.

Macrophages exhibit diverse functions in the tumor microenvironment, many contributing to tumor progression. Tumor-associated macrophages are generally represented by the M2 class of macrophages, distinguished from M1 macrophages by the differential expression of specific cytokines and cell surface markers. To determine the possible role of ezrin in macrophage polarization, we directed the differentiation of ShCtrl and ShEZR THP-1 cells to M0, M1, and M2 sub-classes by specific chemical and cytokine treatments. The ezrin knockdown in THP-1 cells differentiated to M0 with PMA had rather small effects on the mRNA expression of M1 markers *CD80*, *CXCL10* (C-X-C motif chemokine ligand), *IL1B* (interleukin-1β), and *TNF* (tumor necrosis factor-α) (Figure 3A). As expected, differentiation to the M1 phenotype following treatment with interferon-γ and lipopolysaccharide dramatically induced the mRNA expression of all four M1 markers; ezrin knockdown further increased CD80 mRNA expression by about 40% (Figure 3B). Ezrin knockdown in M0 THP-1 cells had little effect on the basal mRNA expression of M2 markers *CD163* and *FN1* (encodes fibronectin), but ~50–60% decreases in *IL10* (interleukin-10) and *CCL22* (C-C motif chemokine ligand 22) mRNA expression were observed (Figure 3C). Differentiation of THP-1 cells to M2 macrophages following treatment with interleukin (IL)-4 and IL-13 markedly increased the mRNA expression of M2 markers (Figure 3D). Importantly, ezrin knockdown cells subjected to differentiation exhibited about a 40–60% reduction of expression of mRNAs encoding two M2 markers—*FN1* and *CCL22*—and a decreasing trend was observed for IL10. The knockdown of moesin did not reprise these results (Figure S1B–E). For example, shRNA-mediated moesin knockdown in M2 THP-1 cells increased the expression of mRNAs encoding the M2 markers *FN1* and *CCL22* suggesting that FERM proteins exhibit differential responses to macrophage polarization (Figure S1E). Recent studies indicate that macrophage differentiation is highly complex, and that the cells can be polarized in a continuum between M1 and M2 subtypes [38]. Our results suggest that macrophage ezrin contributes to the polarization to a more M2-like subtype that can contribute to pro-tumorigenic activity in the tumor microenvironment.

**Figure 4:**
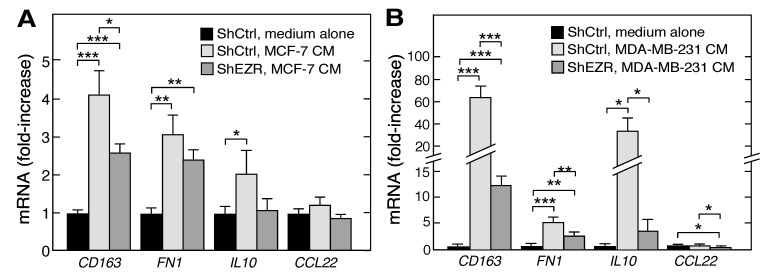
Role of ezrin in tumor cell-driven myeloid cell polarization. (**A**,**B**) ShEZR and ShCtrl cells were incubated with CM from MCF-7 (**A**) or MDA-MB-231 (**B**) cells, or with medium alone, and mRNAs encoding M2 marker proteins determined by RT-qPCR, and normalized to ShCtrl medium alone cells. Mean ± standard deviation; *, **, and *** indicate *p* < 0.05, 0.01, and 0.001, respectively.

Tumor cells can “educate” macrophages for their own advantage, thereby polarizing them towards the M2, pro-tumorigenic phenotype. Here, we investigated the effect of cancer cell-derived CM on macrophage differentiation and on the role of ezrin in this process. CM from both cancer cell lines induced mRNAs encoding the M2 markers *CD163*, *FN1*, and *IL10* in ShCtrl THP-1 cells; the induction ranged from 2- to 4-fold by CM from MCF-7 cells (Figure 4A), and from 5- to 60-fold by CM from MDA-MB-231 cells (Figure 4B). The knockdown of ezrin in THP-1 cells markedly reduced the CM-stimulated expression of the three M2 markers in both cell lines, with a more dramatic influence of MDA-MB-231 CMs (Figure 4A,B). These experiments support and extend our observations of the role of ezrin in macrophage polarization, namely, that the expression of M2 markers is ezrin-dependent.

### 2.4. Macrophage Ezrin Enhances CM-Stimulated Migration, Invasion, and Clonogenic Growth of Tumor Cells

**Figure 5:**
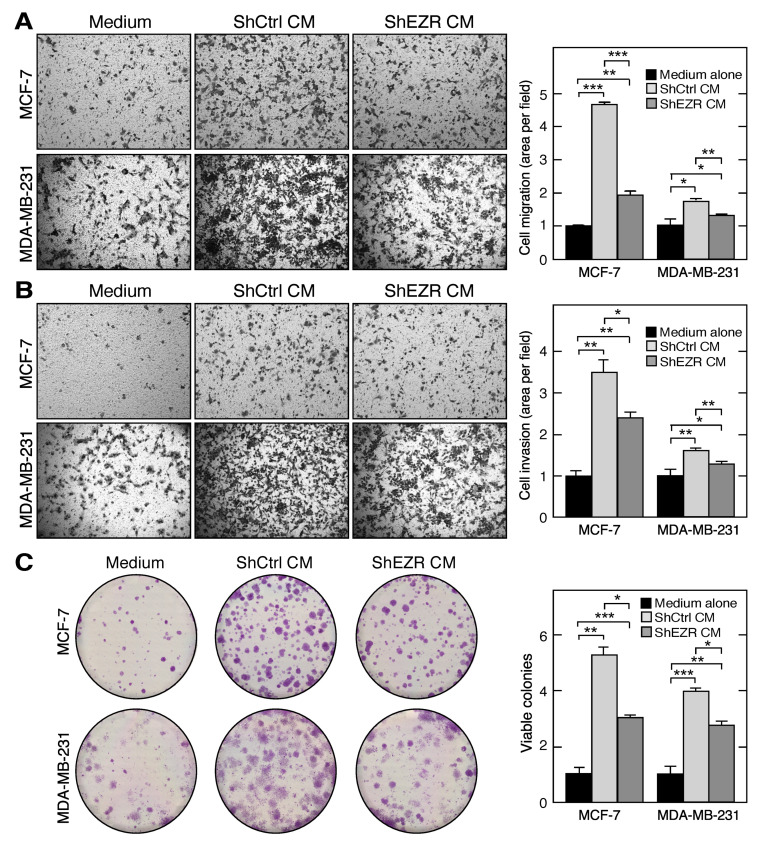
Role of myeloid cell ezrin in tumor cell migration, invasion, and clonogenic growth. (**A**) Trans-well migration of MCF-7 (upper panels) and MDA-MB-231 (lower panels) cells towards the complete RPMI medium (left), CM from M2-polarized ShCTRL (middle) and ShEZR (right) THP-1 cells. Area of transmigrated cells was quantitated (extreme right). (**B**) Matrigel invasion assay with conditions as in (**A**). (**C**) Clonogenic potential of MCF7 and MDA-MB-231 cells were determined in the presence of complete RPMI medium (left), and M2 CM from ShCtrl (middle) and ShEZR THP-1 cells (right). Number of colonies with greater than 50 cells was quantified (extreme right). Mean ± standard deviation; *, **, and *** indicate *p* < 0.05, 0.01, and 0.001, respectively.

Tumor-associated macrophages secrete pro-migratory factors that induce primary tumor cell intravasation, i.e., the invasion of blood vessels, to promote metastasis to secondary sites [39]. In vitro studies have shown that M2 macrophages enhance the migration and invasion of several cancer cell types, including breast cancer. We investigated the influence of macrophage ezrin on the release of factors that induce the migration and invasion of breast cancer cells. Using a trans-well migration assay, M2 CM from ShCtrl THP-1 cells, when used as a chemoattractant, induced the migration of both MCF-7 or MDA-MB-231 cells compared to the complete medium alone (Figure 5A). M2 CM from ShEZR THP-1 cells also induced the migration of both cancer cell lines, compared to the complete medium; however, the extent of migration was reduced compared to ShCtrl CM. Very similar results were observed in an invasion assay in which the trans-well insert was coated with matrigel, and CM from M2 THP-1 cells was used as chemoattractant (Figure 5B).

Tumor growth in vivo can be recapitulated by a clonogenic assay that assesses the in vitro proliferation of cancer cells in the presence of agents from the tumor microenvironment, including tumor-associated macrophage-derived growth factors [40]. To further determine the role of macrophage ezrin in the growth of breast cancer cells, the cells were subjected to clonogenic assay in the presence of CM from ShCtrl or ShEZR M2 THP-1 cells (or complete RPMI medium as the control) for 14 d with medium changes every 3 d. Following fixation and staining, the viable colonies with >50 cells were counted. M2 CM from ShCtrl cells increased the viable colony number in both MCF-7 and MDA-MB-231 cells compared to the medium alone (Figure 5C). The ezrin knockdown of THP-1 cells substantially reduced the M2 CM-stimulated clonogenic growth of both breast cancer cell lines.

### 2.5. Ezrin Enhances Macrophage Angiogenic Potential

**Figure 6:**
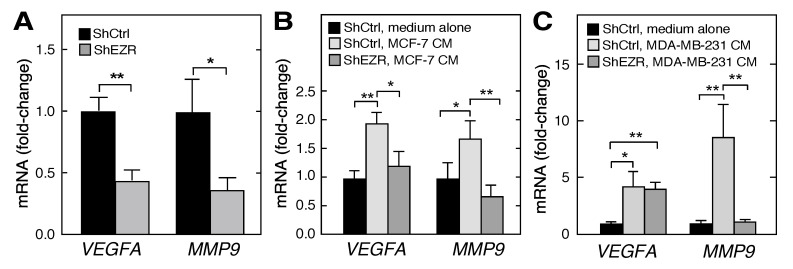
Role of ezrin in the myeloid cell expression of mRNAs encoding tumor growth stimulatory factors. (**A**) *VEGFA* and *MMP9* mRNAs determined in the ShCtrl and ShEZR THP-1 cells by RT-qPCR. (**B**,**C**) ShCtrl and ShEZR THP-1 cells were incubated with CM from MCF-7 (**B**) or MDA-MB-231 (**C**) cells, or with medium alone, and *VEGFA* and *MMP9* mRNAs determined by RT-qPCR; Mean ± standard deviation; * and ** indicate *p* < 0.05 and 0.01, respectively.

Tumor-associated macrophages secrete multiple angiogenic factors, e.g., vascular endothelial growth factor-A (VEGF-A) and matrix metalloproteinase 9 (MMP9), which induce blood vessel initiation and extension, as well as tumor cell metastasis to new sites [41,42]. The basal expression of *VEGFA* and *MMP9* mRNAs was reduced by half or more in ezrin-deficient shEZR THP-1 cells compared to controls (Figure 6A). We investigated the role of ezrin in the tumor cell-directed education of macrophages regarding the angiogenic factor production. The incubation of ShCtrl THP-1 cells with CM from MCF-7 cells nearly doubled the expression of *VEGFA* and *MMP9* mRNAs; however, the expression of both transcripts was reversed to the unstimulated level when ShEZR THP-1 cells were incubated with the CM from MCF-7 cells (Figure 6B). CM from MDA-MB-231 cells induced *VEGFA* and *MMP9* mRNAs by about 4- and 8-fold, respectively (Figure 6C). The induction of *MMP9*, but not *VEGFA*, mRNA was reversed by the ezrin knockdown in ShEZR THP-1 cells. These experiments suggest that macrophage ezrin is a key factor in cell responses to cues in the tumor microenvironment that determines the macrophage secretion of pro-angiogenic factors that contribute to vessel growth and tumorigenesis.

## 3. Discussion

**Figure 7:**
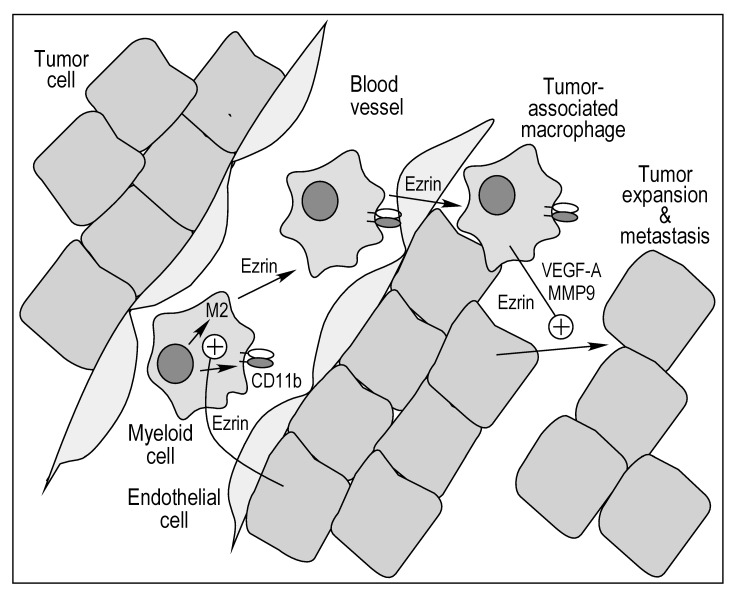
Bidirectional role of ezrin in myeloid and tumor cell interactions in tumorigenesis.

The tumor microenvironment comprises a host of cell types, with myeloid cells occupying up to 25% of the tumor mass [43]. Multiple functions of myeloid cells in tumor initiation, development, and metastasis have been explored in detail [21,44,45]. Here, we investigated the specific role of myeloid cell ezrin in in vitro tumorigenic properties of macrophages, and in the reciprocal communication between these cells and breast cancer-derived cell lines (Figure 7; see Table S1 for summary of results). Leukocyte extravasation into perivascular tissue plays a key role in inflammatory diseases as well as in early stages of tumorigenesis [46]. The initiating step requires leukocyte interaction with vascular endothelium, followed by rolling, arrest, firm adhesion, and ultimately, diapedesis [46]. Importantly, we reveal a critical contribution of myeloid ezrin in endothelial cell interaction as the stable knockdown of ezrin in THP-1 cells abrogates binding to HUVEC by about 75% (Figure 1A). The role of ezrin in THP-1 cell transmigration was determined in a trans-well assay in which the CM from MCF-7 or MDA-MB-231 cells was used as chemoattractant. Ezrin knockdown reduced the stimulated transmigration by about 60% for CM from both cancer cells. Together, these results reveal an unexpected role of myeloid cell ezrin in early stage tumorigenesis. We recognized that macrophages, and their interactions with endothelial cells, have critical functions in a diversity of inflammatory pathologies, e.g., angiogenesis, atherosclerosis, and fibrosis, and likewise, macrophage ezrin might have important functions in multiple pathophysiological conditions besides tumorigenesis [47].

Monocyte/macrophage adhesion to endothelial cells, and the subsequent transmigration, are mediated by the leukocyte integrin, Mac-1, a heterodimeric complex of CD11b and CD18 [37]. The inhibition of CD11b, either by genetic manipulation or by neutralization with anti-CD11b antibody, abrogates leukocyte infiltration into tumors and tumor growth [48,49]. The knockdown of ezrin reduced THP-1 cell expression of *ITGAM* mRNA that encodes CD11b; mRNAs encoding other leukocyte cell surface proteins were unaffected (Figure 2A). We also queried the effect of cancer cells on the expression of *ITGAM* and other mRNAs encoding leukocyte cell surface proteins. Notably, of the seven genes interrogated only *ITGAM* mRNA was consistently induced by CM from both cancer cell types (Figure 2B,C). Furthermore, the induction was completely eradicated in ezrin-depleted THP-1 cells revealing a critical role of ezrin in constitutive and stimulus-inducible *ITGAM* mRNA expression. These results are consistent with previous reports that CD11b is critical for leukocyte binding to endothelial cells and infiltration into tumors [48,49]. However, these results are not without controversy as others have reported myeloid cell invasion into tumors requires the activation of a single integrin, namely, α4β1 [43,50]. Interestingly, the expression of *ITGA4* mRNA, which encodes α4 integrin, is not influenced by ezrin expression (Figure 2).

Tumor macrophages are biased away from the classically activated, pro-inflammatory M1 class, and toward the alternatively activated, immunosuppressive M2 phenotype [36]. The former is considered to be tumor killing, whereas the latter is tumor supportive. However, it is now clear that tumor-associated macrophages are not sub-divided in a binary M1/M2 classification, but rather comprise multiple sub-populations with characteristics of both [45]. The tumor microenvironment reprograms macrophages through the release of metabolites or cytokines, generally inducing polarization towards the M2 phenotype [51]. Our results indicate that macrophage ezrin contributes to tumor microenvironment-directed polarization. The influence of ezrin on the polarization of naïve, M0 macrophages was small (Figure 3A,C). However, polarization towards the M2 class was markedly reduced in ezrin-depleted THP-1 cells as shown by an ~50% reduction in the M2 markers *FN1*, *IL10*, and *CCL22* mRNAs; in contrast, polarization towards M1 was somewhat increased by ezrin depletion (Figure 3B,D). Subsequently, we investigated the role of macrophage ezrin in the tumor cell-mediated reprogramming of polarization. CM from both tumor cell lines induced M2 polarization as shown by the increased expression of *CD163*, *FN1*, and *IL10* mRNAs, and a dramatically reduced expression of these M2 markers was observed in the ezrin-depleted cells (Figure 4). These results reveal an unanticipated role of macrophage ezrin in cell-autonomous and non-autonomous polarization toward the immunosuppressive, tumor-supporting M2 phenotype.

The influence of tumor-associated macrophage-derived factors on tumor cell function and tumor progression is well established [24,45]. Here, we investigated the specific role of ezrin on macrophage communication with tumor cells. We observed that the chemoattraction and matrigel invasion of both breast cancer cell lines toward M2 CM from ezrin-depleted THP-1 cells was markedly reduced compared to control M2 CM (Figure 5). The ability of tumor cells to proliferate in the presence of tumor-associated macrophages under in vitro conditions is a rapid and sensitive surrogate assay for assessing the influence of tumor microenvironment factors and potential therapeutic agents [52]. Colony formation by both MCF-7 and MDA-MB-231 cells was significantly reduced when the cells were incubated with M2 CM from ezrin-depleted THP-1 cells compared to control M2 CM (Figure 5C).

A diverse array of macrophage-derived factors contributes to tumor progression [45]. Among them are growth factors that induce the proliferation of breast cancer cells, including VEGFR2-positive MCF-7 and MDA-MB-231 cell lines [53,54]. In addition, tumor-associated macrophages secrete an active form of MMP-9 that can stimulate tumor cell migration and invasion by the proteolytic cleavage of matrix constituents [55]. The basal expression of *VEGFA* and *MMP9* mRNAs was reduced by about half in the ezrin-depleted THP-1 cells (Figure 6A), comparable to the observed reduction of *ITGAM* mRNA. CM from both breast cancer cell lines increased the expression of both transcripts, and ezrin depletion inhibited the stimulus-dependent expression of *MMP9* mRNA in both cell lines, and *VEGFA* mRNA expression in MCF-7 cells (Figure 6B,C). Intriguingly, ezrin regulates the expression of angiogenic factors in both macrophages as well as tumor cells, as ezrin knockdown in breast cancer cells reduces the macrophage expression of *VEGFA* and *MMP9* mRNAs [56,57]. Thus, ezrin contributes to the basal and stimulus-dependent expression of tumorigenic factors in macrophages, but the nature of the stimulus in cancer cell CM remains to be elucidated.

In several experiments, the functional response of THP-1 cells to CM from MDA-MB-231 cells was considerably greater than the response to MCF-7 CM. For example, the induction of *ITGAM*, *CD163*, *IL10*, *VEGFA*, and *MMP9* mRNAs is up to an order of magnitude greater in the presence of MDA-MB-231 CM compared to MCF-7 CM. These results are consistent with the greater aggressiveness of the former [58]. With a single exception, ezrin knockdown markedly reverses the induction of all transcripts by CM from both cell lines (Figure 5). Complementing the “education” of tumor-associated macrophage gene expression by tumor cells, we show the influence of THP-1 cells on tumor cell chemoattraction, matrigel invasion, and the clonogenic growth of both breast cancer cell lines is in part myeloid cell ezrin-dependent. These bi-directional responses can generate a positive feed-forward loop in which the secretion of factors by tumor cells induces myeloid cell activation and the secretion of myeloid cell factors then further stimulates tumor cells. Most importantly, the dual activation processes are both dependent on myeloid cell ezrin. The integration of these events drives multiple pro-tumorigenic processes including cell adhesion, trans-migration, polarization, and growth factor production. These results also suggest that myeloid cell ezrin is a potential target for therapeutic intervention to reduce breast cancer growth and metastasis.

## 4. Materials and Methods

### 4.1. Cell Culture, Reagents, Constructs, and Antibodies

THP-1, HEK293T, and MDA-MB-231 cells were purchased from ATCC (Manassas, VA, USA) and MCF-7 cells from Sigma-Aldrich (St. Louis, MO, USA). Human umbilical vein endothelial cells (HUVEC) were isolated from human umbilical cords obtained from MetroHealth System (Cleveland, OH, USA). HEK293T cells were grown in DMEM supplemented with 10% fetal bovine serum and 1% penicillin-streptomycin solution, in a humidified 5% CO_2_ chamber. THP-1, MCF-7, and MDA-MB-231 cell lines were cultured in RPMI-1640 medium supplemented with 10% fetal bovine serum and 1% penicillin-streptomycin solution. HUVECs were grown in MCDB 105 medium with 15% fetal bovine serum with 75 mg of endothelial cell growth supplement and sodium heparin isolated from porcine intestine. Calcein-AM was purchased from Thermo-Fisher (Waltham, MA, USA). Ezrin-specific and control shRNAs were from Sigma-Aldrich. Rabbit polyclonal anti-ezrin, anti-moesin, and anti-CD11b antibodies were from Proteintech (Rosemont, IL, USA). Anti-tubulin antibody was purchased from Cell Signaling (Danvers, MA, USA). Goat anti-mouse and anti-rabbit antibodies, and ECL and ECL prime reagents were obtained from GE Healthcare (Chicago, IL, USA). qPCR probes, Halt Protease Inhibitor Cocktail, and One-step Taqman reaction mix were from Thermo-Fisher, and RNA extraction kits were from Zymo Research (Irvine, CA, USA). LipoD93 transfection reagent was purchased from Signagen (Rockville, MD, USA). Amicon Ultra4 concentration tubes were from Millipore-Sigma (Burlington, MA, USA). Puromycin was obtained from Invivogen (San Diego, CA, USA). Phorbol 12-myristate 13-acetate (PMA), polybrene, lipopolysaccharide, and cell lytic buffer were obtained from Sigma-Aldrich. Trans-well chambers were obtained from Corning (Corning, NY, USA). IL-4, IL-13 and interferon-γ were from Peprotech (Rocky Hill, NJ, USA).

### 4.2. shRNA-Mediated Ezrin and Moesin Gene Knockdown

5 × 10^5^ THP-1 cells were transduced with ezrin shRNA and control shRNA lentiviral particles in the presence of polybrene (2 µg/mL) by centrifugation at 1000× g at 32 °C for 30 min. The cell pellet was resuspended in fresh medium and cultured for 72 h before replacement with fresh medium containing puromycin (1 μg/mL). The media were replaced with puromycin-containing medium every 3 days for 3 to 4 weeks for the selection of stably transfected ShEzrin (ShEZR) and ShControl (ShCtrl) cells. Ezrin knockdown was validated by Western blot. An identical procedure was followed for the knockdown of moesin by moesin shRNA (shMSN) in THP-1 cells, as well as validation.

### 4.3. THP-1 Differentiation towards M1 and M2 Subtypes

THP-1 monocytes are differentiated into M0-like macrophages by incubation for 24 h with PMA (150 nM) in RPMI medium. M0-like macrophages were then polarized toward the M1 subtype by 48 h incubation with interferon-γ (20 ng/mL) and lipopolysaccharide (10 pg/mL). For M2 polarization, M0-like macrophages were incubated with 20 ng/mL of IL-4 and 20 ng/mL of IL-13 for 48 h [59].

### 4.4. Preparation of Conditioned Media

For the preparation of the conditioned medium (CM) for the co-culture experiments, 5 × 10^6^ MCF-7 or MDA-MB-231 cells were seeded on 150 mm plates and cultured in complete RPMI medium until about 90% confluent. The cells were washed twice with phosphate-buffered saline (PBS) and refreshed with complete RPMI medium for 24 h. The CM was collected and centrifuged at low speed, passed through a 0.22 μm filter, and stored at −80 °C. To prepare the CM from the M2 macrophages, ezrin knockdown or control THP-1 cells were differentiated to the M2 subtype. Following differentiation, the cells were washed with PBS and incubated with fresh complete RPMI medium for 24 h. The CM was collected, centrifuged at low speed, passed through 0.22 μm filters, and stored at −80 °C.

### 4.5. Monocyte Adhesion and In Vitro Trans-Well Migration Assays

For the adhesion assays, HUVECs were cultured in 6-well plates until 85–90% confluent. THP-1 cells were labeled with calcein-AM (2.5 μM) for 30 min at room temperature and washed once with PBS. Calcein-AM-labeled THP-1 cells (0.5 × 10^6^/mL) were incubated with the HUVEC monolayer for 3 h at 37 °C. The wells were washed twice with PBS to remove unbound or loosely bound THP-1 cells, and luminescence quantified.

A trans-well migration assay was performed in 24-well, trans-well inserts with 5 μm pores (Corning). THP-1 cells were labeled with calcein-AM, and 1 × 10^6^ cells in serum-free RPMI media were added to the trans-well upper chambers. CM from MCF-7 or MDA-MB-231 cells was added to the lower chambers as chemoattractant. After 6 h at 37 °C, the inserts were washed three times with PBS and non-migrating cells removed from the upper chambers by scrubbing with a cotton swab. Migrating cells were imaged at 10X using a Leica DMI6000 inverted microscope with a Leica DFC7000T camera (Leica Microsystems, GmbH, Wetzlar, Germany) and quantified using ImagePro Plus software version 7 (Media Cybernetics, Inc., Rockville, MD, USA).

### 4.6. Treatment of Cells with CM

ShCtrl or ShEZR THP-1 cells (1 × 10^6^) were treated with 150 nM PMA for 24 h to polarize into macrophages. Macrophages were incubated with MCF-7 or MDA-MB-231 CM for 48 h. The cells were washed with PBS and the RNA was isolated.

### 4.7. Semi-Quantitative RT-PCR

Total RNA was extracted from the cells using the RNA isolation kit according to the manufacturer’s protocol. RNA (50 ng per reaction) was subjected to RT-qPCR using gene-specific Taqman probes and one-step Taqman PCR mix. *GAPDH* mRNA expression was used as the internal control.

### 4.8. Western Blot Analysis

Cells were scraped and washed with ice-cold PBS. Following low-speed centrifugation, the pellet was re-suspended in CelLytic M Cell Lysis Reagent for 15 min at 4 °C in the presence of 1X protease inhibitor cocktail, and debris was removed by centrifugation at 15,000 rpm for 15 min at 4 °C. Samples were subjected to SDS-PAGE, and the proteins transferred by electrophoresis to a polyvinylidene fluoride membrane for 45 min at 250 mA. The membrane was blocked with 5% dried non-fat milk in Tris-buffered saline containing 0.05% Tween 20 for 1 h at room temperature, and then incubated overnight at 4 °C with target-specific antibodies. Following three washes with the same buffer, membranes were incubated with horseradish peroxidase-conjugated secondary antibody for 1 h at room temperature. The blots were washed and developed using ECL reagents according to the manufacturer’s protocol.

### 4.9. Cancer Cell Migration and Invasion Assays

Trans-well migration and invasion assay of breast cancer cells was performed using 24-well trans-well chambers with polycarbonate membranes with 8 μm pores (Corning). MCF-7 and MD-MB-231 cells were serum-starved and resuspended in serum-free media. Then, 1 × 10^5^ cells were loaded into the upper chamber and lower chambers contained either compete fresh RPMI-1640 media or M2 CM for ShCtrl or ShEZR THP-1 cells with 10% fetal bovine serum as the chemoattractant. After 24 h, non-migrated cells on the upper surface were removed with a cotton swab; cells migrating to the lower surface were fixed with 100% methanol and stained with 0.5% crystal violet for 2 h, and the images were obtained as above. The total area occupied by migrated cells was quantified using ImagePro Plus version 7 software. For the invasion assay, 2.5 × 10^5^ MCF-7 or MDA-MB-231 cells were used. The assay was done identically to the migration assay except inserts were coated with 1 mg/mL of Matrigel (BD Biosciences, San Jose, CA, USA) and then allowed to polymerize for 2 h at 37 °C before use.

### 4.10. Clonogenesis Growth Assay

To determine the effect of THP-1 M2 CM on the clonogenic growth of MCF-7 and MDA-MB-231 cells, cells (500 per well) were seeded in a 12-well dish and cultured for 14 d in RPMI or in the presence of M2 CM containing 10% fetal bovine serum from ShCtrl and ShEZR THP-1 cells. Medium was replaced with fresh complete medium or CM every 3 d. The cells were fixed with 100% methanol for 15 min followed by staining with 0.5% crystal violet for 2 h. High-resolution images were acquired as above, and colonies of more than 50 cells were counted with Imagepro Plus version 7 software.

### 4.11. Statistical Analysis

All experiments were performed in triplicate, unless otherwise indicated. Data are expressed as the mean ± standard deviation. Statistical analyses were done by Student’s *t*-tests using Prism 7.0 (GraphPad, San Diego, CA, USA). A *p* value < 0.05 was considered statistically significant.

## Supplementary Materials

Supplementary material Figure S1 and Table S1 can be found at https://www.mdpi.com/1422-0067/21/20/7716/s1.

## Abbreviations

MMP	matrix metalloproteinase
VEGF	vascular endothelial growth factor
ITGAM	integrin αM
ERM	Ezrin-radixin-moesin
FERM	four point one and ERM
EC	endothelial cell
EZR	ezrin
MSN	moesin
CXCL	C-X-C motif chemokine ligand
IL	interleukin
CM	conditioned medium
HUVEC	human umbilical vein endothelial cells
TNF	tumor necrosis factor
qPCR	quantitative PCR
