# Supplementary material for: Bidirectional Tumor-Promoting Activities of Macrophage Ezrin
Source: Int J Mol Sci. 2020 Oct 19;21(20):7716. doi: 10.3390/ijms21207716 (PMC7589996; doi:10.3390/ijms21207716)
Supplement: Supplementary file 1 [file ijms-21-07716-s001.pdf]

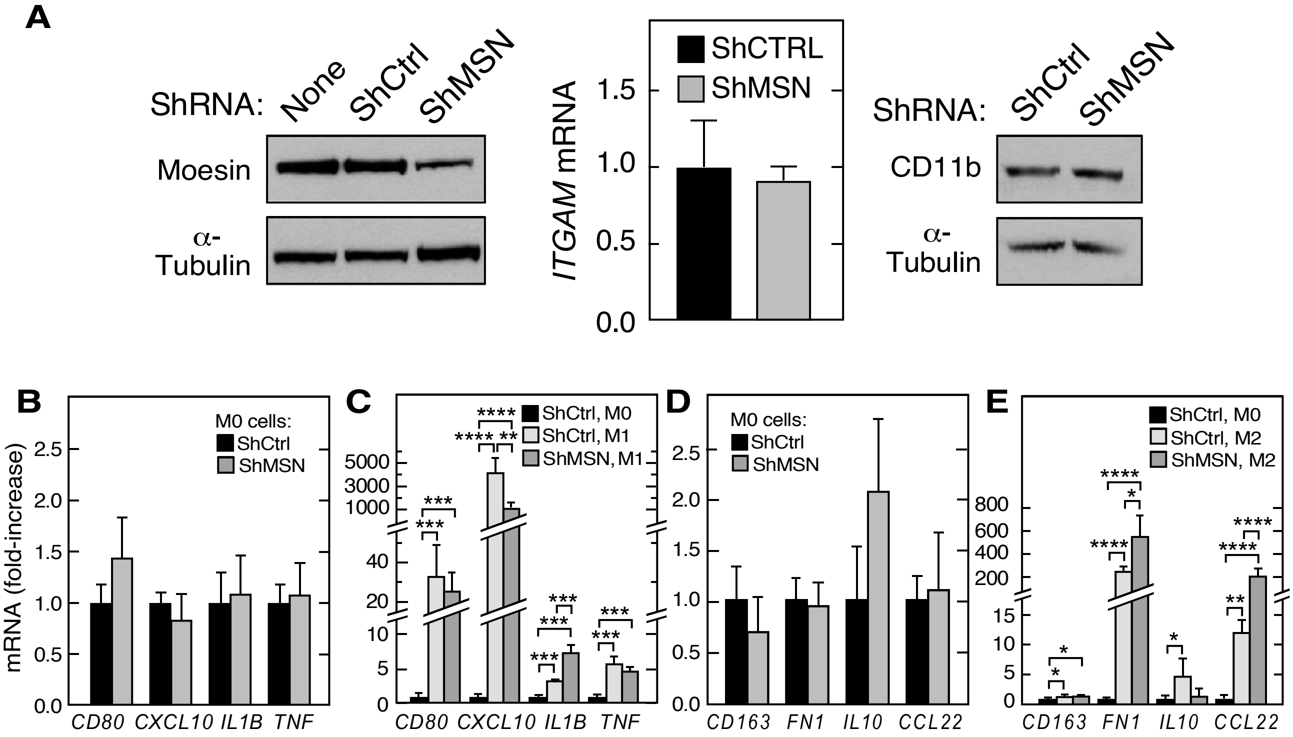

Figure S1. Role of moesin in myeloid cell gene expressiona and polarization. (**A**) Following moesin knockdown in THP-1 cells by moesin shRNA (ShMSN) (left), expression of *ITGAM* mRNA was determined by qPCR (center) and CD11b by immunoblot (right). (**B**) mRNAs encoding M1 marker proteins *CD80*, *CXCL10*, *IL1B*, and *TNF* were determined by RT-qPCR in M0 ShMSN cells and normalized to M0 ShCtrl cells. (**C**) ShMSN cells and ShCtrl were subjected to M1 polarization conditions, and mRNAs encoding M1 marker proteins determined as in (A) and normalized to M0 ShCtrl cells. (**D**) mRNAs encoding M2 marker proteins *CD163*, *FN1*, *IL10*, and *CCL22* were determined by RT-qPCR in M0 ShMSN cells and normalized to M0 ShCtrl cells. (**E**) ShMSN and ShCtrl cells were subjected to M2 polarization conditions and mRNAs encoding M2 markers determined, and normalized to M0 ShCtrl cells. Mean ± standard deviation; *, **, ***, and **** indicate $P < 0.05$, 0.01, 0.001, and 0.0001, respectively.

Table S1. Summary of major results.

THP-1 cell function	Effect of ezrin knockdown
Adhesion to HUVEC monolayer	Reduced
Transmigration towards chemoattractant	Reduced
Expression of chemokines and adhesion receptirs	Pronounced reduction of ITGAM mRNA and CD11b
Macrophage polarization towards M0, M1, and M2 states	Non-significant change towards M0 polarization, but increased M1 and decreased M2 marker expression. Indicates ezrin-dependent expression of pro-tumorigenic state
Secretion of factors that influence breast cancer cell migration and invasion	Reduced
Secretion of factors that influence breast cancer cell clonogenic growth	Reduced
Angiogenic potential	Reduced
